# On the estimation of the effect of weight change on a health outcome using observational data, by utilising the target trial emulation framework

**DOI:** 10.1038/s41366-023-01396-0

**Published:** 2023-10-26

**Authors:** M. Katsoulis, A. G. Lai, D. K. Kipourou, M. Gomes, A. Banerjee, S. Denaxas, R. T. Lumbers, K. Tsilidis, Maria Kostara, A. Belot, C. Dale, R. Sofat, C. Leyrat, H. Hemingway, K. Diaz-Ordaz

**Affiliations:** 1 MRC Unit for Lifelong Health and Ageing, Institute of Cardiovascular Science, University College London, London, UK; 2 Institute of Health Informatics, University College London, London, UK; 3 Inequalities in Cancer Outcomes Network, Department of Non-communicable Disease Epidemiology, London School of Hygiene and Tropical Medicine, London, UK; 4 AstraZeneca, London, UK; 5 Department of Applied Health Research, University College London, London, UK; 6 University College London Hospitals NHS Trust, London, UK; 7 Barts Health NHS Trust, The Royal London Hospital, London, UK; 8 Alan Turing Institute, London, UK; 9 Department of Epidemiology and Biostatistics, School of Public Health, Imperial College London, London, UK; 10 Department of Hygiene and Epidemiology, University of Ioannina School of Medicine, Ioannina, Greece; 11 Department of Pediatrics, University Hospital of Ioannina, Ioannina, Greece; 12 Department of Pharmacology and Therapeutics, University of Liverpool, Liverpool, UK; 13 Department of Medical Statistics, London School of Hygiene and Tropical Medicine, London, UK; 14 Dept of Statistical Science, Faculty of Maths & Physical Sciences, University College London, London, UK

**Keywords:** Epidemiology, Risk factors

## Abstract

**Background/Objectives:**

When studying the effect of weight change between two time points on a health outcome using observational data, two main problems arise initially (i) ‘when is time zero?’ and (ii) ‘which confounders should we account for?’ From the baseline date or the 1st follow-up (when the weight change can be measured)? Different methods have been previously used in the literature that carry different sources of bias and hence produce different results.

**Methods:**

We utilised the target trial emulation framework and considered weight change as a hypothetical intervention. First, we used a simplified example from a hypothetical randomised trial where no modelling is required. Then we simulated data from an observational study where modelling is needed. We demonstrate the problems of each of these methods and suggest a strategy.

**Interventions:**

weight loss/gain vs maintenance.

**Results:**

The recommended method defines time-zero at enrolment, but adjustment for confounders (or exclusion of individuals based on levels of confounders) should be performed both at enrolment and the 1st follow-up.

**Conclusions:**

The implementation of our suggested method [adjusting for (or excluding based on) confounders measured both at baseline and the 1st follow-up] can help researchers attenuate bias by avoiding some common pitfalls. Other methods that have been widely used in the past to estimate the effect of weight change on a health outcome are more biased. However, two issues remain (i) the exposure is not well-defined as there are different ways of changing weight (however we tried to reduce this problem by excluding individuals who develop a chronic disease); and (ii) immortal time bias, which may be small if the time to first follow up is short.

## Introduction

Ideally, to estimate the effect of a weight change intervention in otherwise healthy adults, a randomised control trial (RCT) should be conducted. Individuals with overweight or obesity would be assigned into diet and/or physical activity regimens. Since such RCTs are rare, expensive and difficult to conduct, there is an increasing interest in using observational studies [1–15]. Observational data usually include a larger, more diverse and representative sample, with longer follow up compared to RCTs, and analysis can be conducted in a timely fashion [12–14]. However, the situation is further complicated when using cohorts or Electronic Health Records (EHR), as data on diet are not always recorded. Nevertheless, questions such as ‘What is the effect of bodyweight reduction on cardiovascular disease?’ can still be answered using EHR or data from cohorts [1–14]. Unfortunately, these questions can introduce ambiguity to the definition of ‘baseline’, where eligibility criteria determining who is selected into the study should be met, as well as to the choices of confounders that should be controlled for, i.e. from enrolment or the 1st follow-up?

To help with these complexities, we utilised the framework of target trial emulation (TTE) that has become increasingly popular over the last years [13, 15–18]. Emulating the target trial with observational data involves two broad steps: First, we explicitly specify the protocol of the target trial we want to want to emulate. Second, we mimic each of the component of the target trial utilising our observational data. TTE provides a framework for comparative effectiveness research using healthcare databases or cohort data that offers a number of important advantages for healthcare decision making, especially when timely decisions can save human lives [15]. This framework makes use of the causal inference theory and provides a structured process for the criticism of observational studies, and facilitates researchers to overcome avoidable flaws in the analysis of observational data [18]. This procedure is followed in order to make the results from RCTs and the corresponding observational studies directly comparable. However, in the case of using weight change as exposure, the situation is more complicated.

To illustrate the problem and build intuition we use two examples. First, we present a simplified paradigm from a hypothetical RCT and later we try to assess the treatment effect, using the same individuals identified from a healthcare database. We apply three different methodologies that have been reported in the literature and assess the sources of bias in each case, to make our recommendation. Second, we use a simulation study from a cohort to illustrate the results from the different methods, along with a guidance in the analysis of real-world observational data (in the Appendix) when the exposure is weight change.

### A. Explaining the problem and the challenges in a hypothetical setting in a toy example

In this section, we illustrate the challenges when using weight change as the treatment of interest in a very simple example in which no modelling is required.

#### 1st example: Hypothetical trials

We begin by supposing that the dataset in Table 1 is generated by a hypothetical trial with perfect adherence to the protocol and no loss to follow-up. Both trials enrol individuals aged 45–60 years, free of chronic diseases at baseline. The trial enrols individuals who are overweight (BMI: 25–29.9 kg/m^2^). Participants are randomly assigned into two treatment arms: the 1st arm consists of high intensity physical activity and low caloric intake for 2 years. This dual intervention is expected to result in ~5 kg/m^2^ loss; Therefore, this is considered the weight loss arm of the trial. The 2nd arm includes high intensity physical activity and standard caloric intake for 2 years. This is expected to result in no weight change; hence, this is considered the weight maintenance arm. The protocol allows individuals to abandon their intervention if they develop a chronic condition (e.g. cancer, renal failure etc) during the intervention period (2 years). Individuals are then followed up for a period of time afterwards, in this example for a further 18 years (20 years in total, though in realistic applications this can be much shorter). The endpoint is defined as the occurrence of a cardiovascular disease during the follow-up. For more details on the trial’s protocol, see Table 2. Since our focus is on discussing the sources of systematic biases, rather than statistical considerations relating to estimation and precision, we imagine that we have a very large sample, and in particular, each individual in Table 1 as representing millions of individuals with the same data, so that the 95% confidence intervals(CI) around the point estimates are very narrow. In this simple example, it is clear from Table 1 that there is no difference between the two interventions on CVD after 20 years in the overweight (risk = 3/20 in both interventions).

**Table 1:** The 1st hypothetical dataset from a randomised trial in which 40 people who are overweight^a^ are randomly assigned to one of two interventions: (a) low caloric intake and high physical activity and (b) standard caloric intake and high physical activity.

ID	number of individuals	Intervention^b^	BMI group	*Level of caloric intake*	Physical activity	weight change (in 2 years follow-up)	chronic disease (in 2 years follow-up) affecting weight change	CVD (end of follow-up, i.e. at 20 years)	Risk of developing CVD by intervention (column in italics)
1	1	(a)	overweight	*Low*	high	loss	yes	yes	3/20 = 15% Individuals 1, 3 and 4 developed the outcome (CVD)
2	1	(a)	overweight	*Low*	high	loss	yes	no	
3-4	2	(a)	overweight	*Low*	high	loss	no	yes	
5–20	16	(a)	overweight	*Low*	high	loss	no	no	
21	1	(b)	overweight	*Standard*	high	loss	yes	yes	3/20 = 15% Individuals 21, 23 and 24 developed the outcome (CVD)
22	1	(b)	overweight	*Standard*	high	loss	yes	no	
23–24	2	(b)	overweight	*Standard*	high	remain	no	yes	
25–40	16	(b)	overweight	*Standard*	high	remain	no	no	

The same data were analysed from an observational database, in which data for caloric intake was missing (in italics).

^a^As mentioned in the text, each of these individuals may represent millions (i.e. precision in the confidence interval is beyond the scope of this paper).

^b^Interventions: (a) low caloric intake and high physical activity and (b) standard caloric intake and high physical activity.

**Table 2:** Protocol of the hypothetical trial and the 1st hypothetical observational study.

	Trial that we would like to do but is not feasible	Observational study		
		Methodology 1 (no adjustment for confounders at 1st follow-up)	Methodology 2 (Follow-up starts after weight change)	Methodology 3 (proposed)
Research question^a^	What is the effect of physical activity and caloric intake on CVD	what is the effect of weight change either from a chronic disease or from different hypothetical interventions on CVD	what is the effect of healthy weight change (as a result of different hypothetical interventions) on CVD	
Eligibility criteria	Trials would enrol otherwise healthy individuals at baseline, aged 45–60yo. Individuals with prevalent CVD or other chronic diseases at date of enrolment are exclude. The trials will be conducted in overweight individuals.	Same criteria applied at enrolment	Same criteria applied at enrolment. Individuals should not develop the outcome or any other chronic disease between the enrolment and the 1st follow-up	Same criteria applied at enrolment. Individuals should not develop the outcome or any other chronic disease between the enrolment and the 1st follow-up
Treatment strategies	a) High physical activity and moderate caloric intake (expecting result: weight loss of 5 kg/m^2^ in 2 years) b) High physical activity and high caloric intake (expecting result: weight maintenance in 2 years) These interventions would last for 2 years.	a) weight loss b) weight maintenance These interventions would last for 2 years.		
Time zero (beginning of follow-up)	At enrolment	At enrolment	At 1st follow-up	At enrolment
Allocation to interventions^b^	Physical activity and caloric intake are used at enrolment	Weight (or BMI) is used at enrolment and weight change measured at the 1st follow-up	Weight (or BMI)is used at enrolment and weight change measured at the 1st follow-up	Weight (or BMI)is used at enrolment and weight change measured at the 1st follow-up
Assignment procedures	Random assignment to treatment strategy	Non-randomly assigned to a weight change intervention		
Causal contrast	Intention-to-treat^c^	Observational analogue of the per-protocol effect		
Follow-up	20 years	20 years	18 years	20 years
Outcome	Fatal and non-fatal CVD	Same	Same	Same
Statistical analysis	None	Apply exclusion criteria at enrolment. Adjust for confounders measured at enrolment in the outcome regression models (in our example from Table 1, we have no such confounders).	Apply exclusion criteria at 1st follow-up. Adjust for confounders measured at the 1st follow up in the outcome regression models.	Apply exclusion criteria both at baseline and 1st follow-up. Adjust for confounders measured both at enrolment and the 1st follow-up in the outcome regression models.

^a^The research question reflects the estimand in these studies, i.e. the quantity we aim to measure.

^b^The allocation to a specific intervention is based on baseline definition.

^c^In this example, we have perfect adherence, so the intention-to-treat effect is also the per-protocol effect.

#### The same dataset from a healthcare database

Now, let us assume that we would like to conduct the same analysis using a cohort from a healthcare database, which has exactly the same people with the above-mentioned hypothetical trial (Table 1). Since typically, most of the available healthcare databases have information on physical activity, but relatively few have information on diet, we assume that all the information on dietary intake is not available for our observational analysis (highlighted in italics in Table 1). That being the case, we now focus on the effect of a ‘proxy intervention’ [19], i.e. ‘healthy’ weight change from enrolment to the 1st follow-up after two years instead of the effect of physical activity and diet. However, there are two extra problems, compared to the usual analysis of observational data; (i) weight change is not a well-defined intervention [12, 13, 20, 21] and (ii) the definition of ‘baseline’ is non-trivial, which subsequently complicates the analysis plan for the selection of confounders.

To further simplify the exposition, consider a situation where the only factor that affects weight change between baseline and the 1st follow-up, apart from physical activity and diet, is the occurrence of a chronic disease [either the outcome (CVD) or other chronic conditions like cancer, diabetes, etc], see Table S1 (Appendix).

**Fig. 1:**
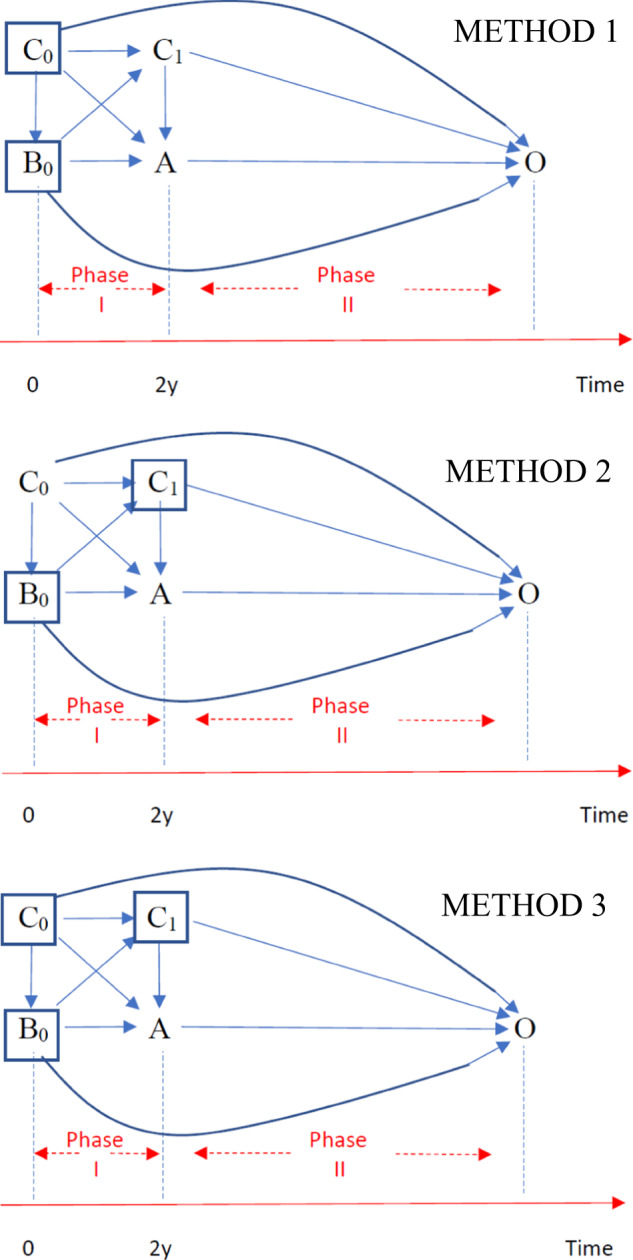
Directed acyclic graphs for the effect of weight change on CVD, using 3 different methods. METHOD 1 (Upper Panel): B_0_ is weight at time 0 and B_1_ is weight at 2 years. C_0_ are the confounders at time 0 and C_1_ are the confounders at 2 years and O is the outcome. Time is split into two phases. Phase I is between weight measurements at time 0 and the 1st follow-up. Phase II is after the weight measurement at the 1st follow-up weight change is only observed at 2 years (end of Phase I), i.e. when we can measure A = B_1_-B_0_. A and B_1_ can be used in the DAG interchangeably, as they are deterministically related. Our aim is to find the effect of A (weight change) on the outcome O. From this DAG, we stratify for B_0_ and we control for C_0_. However, we do not control for C_1_ (or exclude individuals based on levels of chronic disease at the 1st follow-up). Our aim is to find the effect of A (weight change) on the outcome O. If we do not control for C_1_, we leave the backdoor path A<--C_1_-->O open. METHOD 2 (Middle Panel): B_0_ is weight at time 0 and B_1_ is weight at 2 years. C_0_ are the confounders at time 0 and C_1_ are the confounders at 2 years and O is the outcome. Time is split into two phases. Phase I is between weight measurements at time 0 and the 1st follow-up. Phase II is after the weight measurement at the 1st follow-up. weight change is only observed at 2 years (end of Phase I), i.e. when we can measure A = B_1_–B_0_. Our aim is to find the effect of A (weight change) on the outcome O. From this DAG, we stratify for B_0_ and we control for C_1_. However, we do not control for C_0_ (or exclude individuals based on levels of chronic disease at the time 0). If we do not control for C_0_, we leave the backdoor path A<--C_0_-->O open. METHOD 3 (Lower Panel): B_0_ is weight at time 0 and B_1_ is weight at the 1st follow-up (at 2 years). C_0_ are the confounders at time 0 and C_1_ are the confounders at 2 years and O is the outcome. Time is split into two phases. Phase I is between weight measurements at time 0 and the 1st follow-up. Phase II is after the weight measurement at the 1st follow-up. Weight change is only observed at the 1st follow-up (end of Phase I), i.e. when we can measure A = B_1_–B_0_. A and B_1_ can be used in the DAG interchangeably, as they are deterministically related. Our aim is to find the effect of A (weight change) on the outcome O. From this DAG, we stratify for B_0_ and we control for C_0_ and C_1_. In other words, there is no backdoor path open.

Next, we analyse these data using three different methods that have been widely used in the literature to estimate the effect of weight change and highlight the pitfalls and the differences in the analysis. We explain in detail these methods using Directed Acyclic Graphs (DAGs) in Fig. 1 and Fig. S1 (Appendix). We present the protocol of the hypothetical trial and the observational study in Table 2 and the results in Table S2 (Appendix).

### Different methods used in the literature

*Method 1*

*a. Baseline definition: Time zero is defined at enrolment*.

*b. Baseline confounders: Adjustment/stratification on initial weight (or BMI) is considered at enrolment. Adjustment/stratification on other confounders measured at enrolment.*

**Fig. 2:**
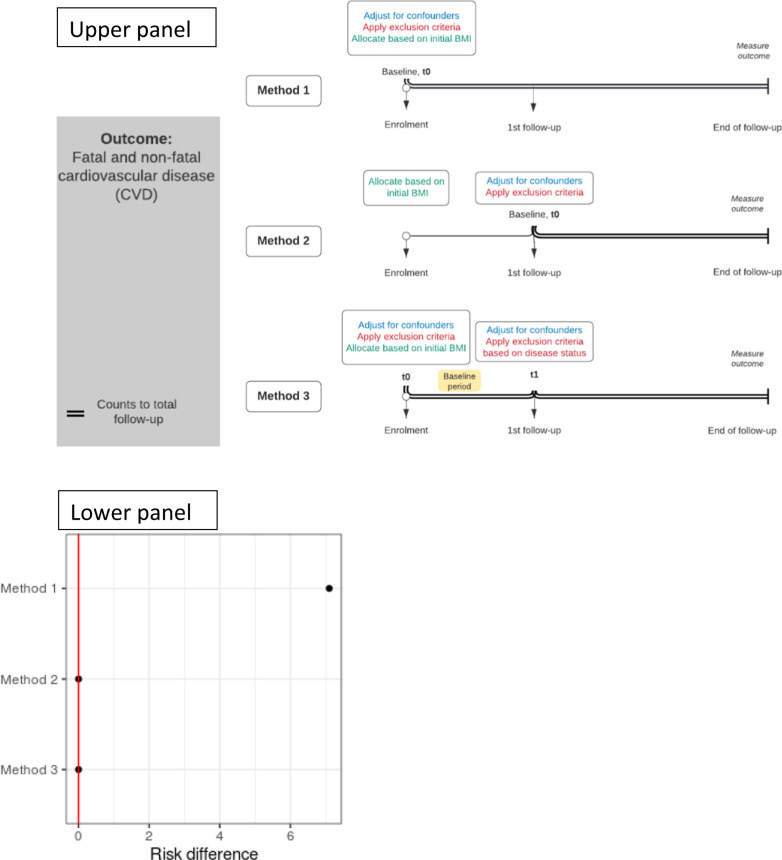
Summary of different methods used to estimate the effect of weight change on CVD from an observational study and results from the 1st example. Upper panel: definition of baseline, adjustment for baseline confounders, criteria for individuals’ allocation to specific groups as well as for exclusion from the study. Lower panel: estimation of risk difference of weight loss vs maintenance on fatal and non-fatal CVD from the 1st toy example (see Table 1), when using different methods to estimate the effect of BMI change, and comparison^†^ with the corresponding risk difference of the interventions in the trial (red line). ^†^This comparison is not well defined, because the estimand (i.e., the quantity we aim to estimate) is different in the analysis of the trial and the observational database, even if we talk for the same individuals. In the trial, we measure the effect of physical activity and diet on CVD, while from the observational data, the quantity of interest is the effect of weight change on CVD. However, in our oversimplistic scenario, in which weight change can be caused either by physical activity, diet or a chronic disease (i.e., no individual on orlistat or other drugs, no individual on chopped off her arms, etc.), and all individuals had the same levels of physical activity and we can account for (i.e., exclude individual with) chronic disease in the analysis of observational data, then the weight loss and weight maintenance arm of the trial are closely related to the weight change from the observational data.

This approach (Method 1) implicitly assumes [1–3] that since time zero is defined at enrolment, all confounders can be accounted for either by adjusting for them or excluding participants based on the existence of specific chronic diseases at enrolment according to the eligibility criteria (see Fig. 2). This would be the correct strategy for time-fixed (single time-point) interventions (e.g. effect of bariatric surgery [4]). However, in this example, using method 1, the exposure (weight change) has not been observed yet (as we can measure weight change only at the 1st follow-up) and the estimand (i.e. the quantity we aim to estimate) here is ‘the effect of weight change either from a chronic disease or from different hypothetical interventions’, see Fig. 1 (upper panel). From this question, we obtain completely different results, compared to the trials’ results (see the research questions from the trial as well as from the analysis of the healthcare database using different methods in Table 2).

Instead of estimating a null effect in the overweight (result from the RCT), this method obtains a risk difference (weight loss vs maintenance) equal to 7.1%. This difference in the findings occurred because this strategy fails to take into account that people’s observed weight loss might be due to a chronic disease between the chosen time zero and the 1st follow-up [See Fig. 1 (upper panel) and Table S3].

*Method 2*

*a. Baseline definition: Time zero is defined at the* 1st *follow-up*.

*b. Baseline confounders: Adjustment/stratification on initial weight (or BMI) is considered at the enrolment. Adjustment/stratification on other confounders measured at the* 1st *follow-up.*

Given that time zero is defined 2 years after the enrolment, in these settings, the follow-up time will be 18 years (20−2 = 18 years). This approach [9–11] has many advantages compared to the previous one (Method 1). First, this strategy correctly excludes individuals who developed chronic diseases from enrolment till the end of the 1st follow-up, because these diseases might have influenced weight change. Moreover, we have successfully allocated individuals to a weight change group based on their initial weight (or BMI) at enrolment and their observed weight change trajectory (from enrolment until the 1st follow-up). In Table S2 (Appendix), using this method, we estimate a risk of 2/18 in both weight loss and maintenance, in other words, no risk difference between weight loss and maintenance (as in the trial). However, the absolute risks were different compared to the trial [2/18 = 11.1% using method 2 in the observational data (Table S2, Appendix) vs 3/20 = 15% in the trial (Table 1)].

Nevertheless, the concern is that time-zero (1st follow-up) is not aligned with the beginning of the exposure (enrolment). This method has led many researchers to adjust for the participant’s characteristics (apart from weight or BMI at enrolment) corresponding to the time of the 1st follow-up, which is considered time zero [9–11]. Using this approach, usually these studies ignore other variables occurring at or before enrolment, but are indeed associated with weight change and the outcome [Fig. 1 (middle panel)]. We see that when comparing the risk differences from the observational study and the trial, the estimates are the same. In more realistic scenarios, when modelling is required (presented later in this paper), the bias can be more pronounced (here the only confounders have been considered chronic diseases and at time zero, everybody was disease free), because there is a backdoor path open from weight change, i.e exposure <-- confounders at time zero --> outcome, see Fig. 1 (middle panel).

*Method 3 (PROPOSED)*

*a. Baseline definition: Time zero is defined at enrolment, at the beginning of the (hypothetical) intervention*.

*b. Baseline confounders: Adjustment/stratification on initial weight (or BMI) is considered at enrolment. Adjustment/stratification on other confounders at enrolment and at the* 1st *follow-up. That is, first, we adjust for confounders at time zero to emulate randomisation at enrolment and exclude individuals based on the eligibility criteria at enrolment. We additionally adjust for confounders (or exclude individuals based on their levels of confounders) measured at the* 1st *follow-up to account for confounders of weight change.*

This is the method we advocate for. It has been used on more recent papers that use the causal inference framework to mimic hypothetical interventions [12–14] and is the one we recommend. These studies usually focus on sustained interventions over time (not only till the 1st follow-up), however, in this paper, we just adopt their definition of baseline.

Using this method, we estimate no risk difference between weight loss and maintenance (as in the trial, see Fig. 2 and Table S2 in the Appendix), even if the absolute risks were different compared to the trial [2/18 = 11.1% using method 3 in the observational data (Table S2, Appendix) vs 3/20 = 15% in the trial (Table 1)]. The main difference compared to method 2 in this example is the duration of the follow-up time, which is 18 years in method 2 but 20 years in method 3, as it begins at enrolment. We should exclude individuals that develop the outcome, or any other chronic disease between enrolment and follow-up to ensure that these diseases are not the cause of the weight change. This also alleviates to a certain extend the issue of the exposure (weight change) being somewhat ill-defined, (by excluding ‘unhealthy’ weight change), see Fig. 1 (lower panel). This estimand only applies to people experiencing ‘healthy’ weight change, without developing the outcome or a chronic disease during the first 2 years. In our toy example we have only one confounder (chronic disease at 1st follow-up) based on the values of which we exclude individuals. Hence, in this simplistic example, where we compute the risks without modelling and we exclude patients developing a chronic disease between the enrolment and the 1st follow-up (the only confounding factor here), the estimates are identical to those obtained by method 2. In a more realistic setting, we additionally need to adjust for other confounders in an outcome regression models [13] (see example 2 below). Accounting for confounders at enrolment is performed to emulate randomisation at enrolment and adjustment for confounders at the 1st follow-up will account for confounders of weight change. Additionally, we exclude individuals based on their health status between the enrolment and the 1st follow-up to define our exposure as ‘healthy weight change’ and make the hypothetical weight change interventions less ill-defined [13, 20, 21].

Usually, we should adjust for (or exclude individuals based on) confounders measured at enrolment and the 1st follow-up, when the research question is related to (healthy) weight change in healthy individuals at enrolment [13]. In this case, we define a baseline period between enrolment and the 1st follow-up, during which the eligibility criteria for chronic diseases is extended from baseline till the end of the 1st follow-up. This, however, might not always be the case. If for example, the research question is for the effect of (healthy) weight change in individuals with cancer at enrolment, we will then include individuals with cancer at enrolment but we will exclude those who developed e.g. diabetes between the enrolment and the first follow-up.

Our recommended approach has two advantages, compared to methodology 2:

- It is consistent with the timing of the interventions. Time zero is defined at the beginning of the interventions (i.e. at enrolment).
- Most importantly, to emulate randomisation at baseline using method 3 in cohort databases or EHR, there is a potential risk that the adjustment for ‘baseline’ confounders is not clear and adequate. This will be demonstrated in the next example (simulation study; see below), in which modelling is required.

While this is the preferred method, it suffers from immortal time bias [16, 22, 23] as a result of all individuals needing to survive and remain healthy from enrolment until the first follow up period, to be included in the study. Immortal time bias can sometimes be accounted for through ‘cloning, censoring, and weighting’ (or through randomly assigning the individual to one of the strategies) [16, 23] when individuals are compatible with multiple interventions from time zero till the point they remain immortal. Unfortunately, this technique cannot be applied here, where the treatment strategy is only known at the first follow-up period (i.e. after it has happened), as this means that individuals can only be compatible with one hypothetical intervention at baseline, so that they end up either losing or maintaining weight. The immortal time bias resulting from this method is not expected to be substantial, if (a) the chronic diseases occurred from the enrolment till the 1st follow-up are not caused by the weight change interventions, (b) the period between enrolment and the 1st follow-up is relatively small, compared to total follow-up time and (c) the incidence of chronic diseases between enrolment and the 1st follow-up is relatively small.

*Assumptions needed to estimate* *the causal effect of lifestyle interventions through using only the proxy exposure ‘weight change’*: Apart from the usual assumptions needed to estimate a causal effect (i.e. positivity, consistency and conditional exchangeability), in case we want to estimate the effect of lifestyle interventions on a health outcome, we need to consider an extra assumption, ‘proxy separation’ [19]. This is a strong assumption, under which all individuals who experienced weight loss, should have been under diet with low caloric intake and increased levels of physical activity and all individuals who experienced weight maintenance, should have been under diet with standard caloric intake and increased levels of physical activity. This condition held in this example, but in real life examples, it is more reasonable to assume that this extra assumption (proxy separation), along with positivity, consistency, conditional exchangeability might hold, at best, approximately [19].

### Other methodologies in the literature

There have also been papers that have combined methods 2 and 3, e.g. in the analysis of Wannamethee et al. [24], the authors practically used method 2 but adjusted for one confounder from time zero, smoking status (like in method 3). Moreover, some papers [5–8] have set baseline at the time of the 1st follow-up and have considered BMI change amongst individuals at a certain BMI group, measured after the (hypothetical) intervention, i.e. at the 1st follow-up visit. In other words, the research question this method addresses is awkward, i.e. ‘what is the effect of healthy weight change among individuals at a given BMI group measured after weight change’, which is definitely not suitable to estimate the causal effect of weight change [5–8].

### B. Presentation of the same problem in a simulation study

We now use a simulated cohort study where modelling is required to present some of the challenges when estimating the effect of weight change using the three methods described previously.

#### Simulated cohort study

We simulated 10,000 individuals who are overweight (BMI: 25–29.9 kg/m^2^), with an overall follow-up time of 20 years. We assumed that individuals had follow-up visits every 2 years. The outcome was CVD occurrence and for simplicity, we assumed no individual died before developing the outcome. The question of interest was the effect of weight change (loss: <5%, maintenance: ≥5% & ≤5% and gain: >5%) on CVD onset. The dataset was simulated so that there is a) no effect of weight loss (vs maintenance) on CVD onset (log HR = 0) and b) log HR of weight gain (vs maintenance) = 0.3. We additionally simulated age, sex, family history of CVD, weight at enrolment and two confounders measured both at enrolment and at 1st follow-up time (smoking status and use of diuretics). For more details about the dataset, see Appendix (Section 2).

We used pooled logistic regression to estimate the effect of weight change on CVD onset. In method 1, we adjusted for confounders at enrolment only (age, sex, family history of CVD, weight at enrolment, smoking status and diuretics) and we did not exclude CVD cases occurred during the first 2 years. In method 2, we adjusted age, sex, family history of CVD, weight at enrolment and confounders measured 2 years after enrolment only (smoking status and diuretics) and excluded CVD cases occurred during the first two years. In method 3, we adjusted for age, sex, family history of CVD, weight at enrolment and confounders measured both at enrolment (smoking status and diuretics) and two years after enrolment (smoking cessation and diuretics) and excluded CVD cases occurred during the first two years.

In this analysis, one time point corresponds to 2 years, and hence the exposure ‘weight change’ corresponds to the change from enrolment to the 1st time point (see https://github.com/mkatsoulis82/On-the-estimation-of-the-effect-of-weight-change-on-a-health-outcome). In Table 3, we estimated the log-hazard ratios using all three methods. We observe that the optimal method to estimate the effect of weight change on CVD is method 3. For the estimation of the effect of weight loss vs maintenance, the bias of method 3 was 0.00 (i.e. estimated logHR = true logHR = 0) and the CI coverage was 95.4% (i.e. very close to 95%). Moreover, method 2 was better (bias = 0.11, CI coverage = 80.4%) compared to method 1 (bias = 0.36, CI coverage = 1.5%) but worse vs method 3. For the relationship of weight gain vs maintenance, the bias of method 3 was 0.00 (i.e. estimated logHR = true logHR = 0.3) and the CI coverage was 95.1% (i.e. very close to 95%). Additionally, method 2 was better (bias = −0.05, CI coverage = 92.0%) compared to method 1 (bias = −0.29, CI coverage = 5.4%) but worse vs method 3.

**Table 3:** Simulation results: Estimating bias and coverage percentage of log hazard ratios of weight loss vs maintenance on CVD from the 2nd hypothetical example in overweight individuals, when using pooled logistic regression and different methods to estimate the effect of weight change.

	True value	Method 1^a^			Method 2^b^			Method 3^c^		
		Estimate	Bias	Confidence interval coverage	Estimate	Bias	Confidence interval coverage	Estimate	Bias	Confidence interval coverage
Log(HR)^d^ Weight loss vs maintenance	0	0.36	0.36	0.015	0.11	0.11	0.804	0.00	0.00	0.954
Log(HR)^d^ Weight gain vs maintenance	0.30	0.01	−0.29	0.054	0.25	−0.05	0.920	0.30	0.00	0.951
Follow-up	20 years	20 years			18 years			20 years^e^		

^a^Method 1: Adjustment for age, sex, family history of CVD, weight at enrolment and confounders measured at enrolment only (smoking status and diuretics). Not excluded CVD cases occurred during the first 2 years. Total sample size: 10,000. Time zero: Enrolment. Total follow-up time:20 years.

^b^Method 2: Adjustment for age, sex, family history of CVD, weight at enrolment and confounders measured at 1st follow-up time (smoking status and diuretics). Excluded CVD cases occurred during the first 2 years. Total sample size: 9731. Time zero: First follow-up. Total follow-up time:18 years.

^c^Method 3: Adjustment for age, sex, family history of CVD, weight at enrolment and confounders measured both at enrolment (smoking status and diuretics) and at 1st follow-up time (smoking cessation and diuretics). Excluded CVD cases occurred in the first 2 years. Total sample size: 9731. Time zero: Enrolment. Total follow-up time: Baseline period + modelling period = 20 years.

^d^The log-hazard ratio estimates were approximately the same when using Cox regression instead of pooled logistic regression.

^e^2 years of baseline period and 18 years of modelling period.

As before, the first difference between methods 2 and 3 in this example is that the total follow-up time is 18 years using method 2 and 20 years using method 3 [18 years of the modelling period plus 2 more years of a period with zero risk (baseline period)]. The second difference is the variables used to adjustment for confounding, with method 3 using variables at enrolment and first visit, while method 2 only uses first visit. For more details, see Appendix, Section 2. We also provide further details on how to estimate weighted Kaplan-Meier curves using IPW in Section 3 of the Appendix and incidence risk curves using the g-formula and pooled logistic regression in Section 4 of the Appendix.

## Discussion

In this article, we highlighted the challenges arising when estimating the effect of weight change on a health outcome. Researchers have used different methodologies for defining time-zero, deciding which confounder measurements to adjust for as well as the defining the eligibility criteria for inclusion [1–14]. The different methods have different sources of bias and some lead to different estimands (see Table 2 and Table S3), thus making the meta-analysis estimates of these studies highly uninterpretable [25–29]. We assessed these biases and the problematic interpretations from the analysis using three different methodologies and illustrated these with numerical examples. Method 3, which has been recently proposed within a causal inference framework to emulate hypothetical interventions [12–14], is the most adequate as it avoids most of the biases and therefore should be preferred. In this method, time zero is set at enrolment and adjustment for confounders should be performed at time zero to emulate randomisation at baseline and exclude individuals based on the eligibility criteria at enrolment. Additionally, adjustment for confounders (or exclude individuals based on their levels of confounders) measured between the enrolment and the 1st follow-up should be applied to make the hypothetical interventions more well-defined.

In summary, methods 1 and 2 are more biased when the research question is the estimation of the effect of (healthy) weight change on a health outcome (e.g. CVD). Compared to our recommended approach (i.e. method 3), method 2 is more biased because it fails to take into consideration the confounders measured at time 0. Nevertheless, it is less biased than method 1, as by adjusting for confounders at the 1st follow-up, we (implicitly) incorporate most of the information of the same confounders at time 0, because usually some of these confounders are the same (i.e. sociodemographic factors) or some lifestyle factors that do not change substantially (for most participants) between baseline and the 1st follow-up (e.g. smoking status). The problems of the different methods are summarised in Table S3 (Appendix).

### Strengths and limitations

Our recommended method 3 enables researchers to minimise the bias from residual confounding by adjusting for confounders measured both before enrolment and during the period where the exposure is not yet observed (between enrolment and 1st follow-up). Researchers following method 2 usually adjust for confounders only at the time of the 1st follow-up [9–11]. Moreover, the method we are advocating for (Method 3) is consistent with the timing of the interventions as time zero is defined at the beginning of the interventions.

This study has some limitations. Weight change is not a well-defined intervention [13, 20, 21]. In other words, we do not know how people lost, maintained or gained weight (consistency assumption) when using observational data. We tried to make our intervention less ‘ill-defined’ (by accounting for healthy weight change) through excluding individuals whose weight change was a result of a disease that occurred between enrolment and the 1st follow-up. Moreover, it should be noted that the results from the observational data from all these methods might differ from the results of the (hypothetical) trial, because the estimand is different. In the trials, the causal contrast is the comparison of well-defined interventions, defined by physical activity and caloric intake. On the other hand, when using observational data without sufficient information of weight change factors (e.g. physical activity, diet) the research question is about weight change, which is a consequence of hypothetical interventions. Additionally, we assumed there is no information that allows us to distinguish intentional from unintentional weight changes.

As we mentioned before, our recommended method can suffer from immortal time bias. In other settings, this can be eliminated by ‘cloning, censoring, and weighting’ (or through randomly assigning the individual to one of the strategies) [16, 23]. However, this cannot occur in the problem of weight change, as this means that individuals can only be compatible with one hypothetical intervention at baseline, so that they end up either losing or maintaining weight. Moreover, the same problem for the baseline definition and baseline confounders arises in more complex scenarios, where the research question is about sustained interventions over time where the g-methods should be used to account for time-dependent confounding [12–14]. Additionally, when we want to estimate the effect of lifestyle interventions on a health outcome through weight change (which is a proxy intervention [19]), we need to consider an extra assumption, ‘proxy separation’.

Finally, in this paper, we discussed only hypothetical interventions that are used in obesity research [1–14] to tackle the obesity epidemic [30, 31]. However, the problems appear in other settings, when the exposure is defined as a change in risk factor in general, e.g. systolic blood pressure [17], heart rate [32], physical activity [33] etc.

## Conclusion

When the exposure of interest is weight change, time zero should be defined at enrolment. Adjustment for confounders measured at enrolment and exclusion of individuals based on the eligibility criteria at enrolment should be performed to emulate randomisation at enrolment. Additionally, exclusion of individuals based on their levels of confounders measured at the 1st follow-up should be applied to make the hypothetical interventions less ill-defined [13, 20, 21] and adjustment for confounders at the 1st follow-up will account for confounders of weight change. Notably, our study demonstrates that this methodology yields much smaller biases compared to other approaches when estimating the effect of weight change. The findings of our paper will also benefit researchers aiming to conduct meta-analyses on weight (or BMI) change [25–29]. It is important for researchers to consider the methodologies employed in different papers to avoid combining dissimilar approaches and drawing inaccurate conclusions.

### Supplementary information

Appendix

## References

[1] Bamia C, Halkjaer J, Lagiou P, Trichopoulos D, Tjønneland A, Berentzen TL (2010). Weight change in later life and risk of death amongst the elderly: the European prospective investigation into cancer and nutrition-elderly network on ageing and health study. J Intern Med.

[2] Dahl AK, Fauth EB, Ernsth-Bravell M, Hassing LB, Ram N, Gerstof D (2013). Body mass index, change in body mass index, and survival in old and very old persons. J Am Geriatr Soc.

[3] Newman AB, Yanez D, Harris T, Duxbury A, Enright PL, Fried LP (2001). Cardiovascular Study Research Group. Weight change in old age and its association with mortality. J Am Geriatr Soc.

[4] Douglas IJ, Bhaskaran K, Batterham RL, Smeeth L (2015). Bariatric Surgery in the United Kingdom: a cohort study of weight loss and clinical outcomes in routine clinical care. PLoS Med.

[5] French SA, Folsom AR, Jeffery RW, Williamson DF (1999). Prospective study of intentionality of weight loss and mortality in older women: the Iowa Women’s Health Study. Am J Epidemiol.

[6] Locher JL, Roth DL, Ritchie CS, Cox K, Sawyer P, Bodner EV (2007). Body mass index, weight loss, and mortality in community-dwelling older adults. J Gerontol A Biol Sci Med Sci.

[7] Sares-Jäske L, Knekt P, Eranti A, Kaartinen NE, Heliövaara M, Männistö S (2020). Intentional weight loss as a predictor of type 2 diabetes occurrence in a general adult population. BMJ Open Diab Res Care.

[8] Gregg EW, Gerzoff RB, Thompson TJ, Williamson DF (2004). Trying to lose weight, losing weight, and 9-year mortality in overweight U.S. adults with diabetes. Diabetes Care.

[9] Williamson DF, Pamuk E, Thun M, Flanders D, Byers T, Heath C (1999). Prospective study of intentional weight loss and mortality in overweight white men aged 40–64 years. Am J Epidemiol.

[10] Mikkelsen KL, Heitmann BL, Keiding N, Sorensen TI (1999). Independent effects of stable and changing body weight on total mortality. Epidemiology..

[11] Yuan Y, Liu K, Zheng M, Chen S, Wang H, Jiang Q (2022). Analysis of changes in weight, waist circumference, or both, and all-cause mortality in Chinese adults. JAMA Netw Open.

[12] Vangen-Lønne AM, Ueda P, Gulayin P, Wilsgaard T, Mathiesen EB, Danaei G (2018). Hypothetical interventions to prevent stroke: an application of the parametric g-formula to a healthy middle-aged population. Eur J Epidemiol.

[13] Katsoulis M, DeStavola B, Diaz-Ordaz K, Gomes M, Lai A, Lagiou P (2021). Weight change and the onset of cardiovascular diseases: emulating trials using electronic health records. Epidemiology..

[14] Jain P, Suemoto CK, Rexrode K, Manson JE, Robins JM, Hernán MA (2020). Hypothetical lifestyle strategies in middle-aged women and the long-term risk of stroke. Stroke..

[15] Hernán MA, Robins JM (2016). Using big data to emulate a target trial when a randomized trial is not available. Am J Epidemiol.

[16] Hernán MA, Sauer BC, Hernández-Díaz S, Platt R, Shrier I (2016). Specifying a target trial prevents immortal time bias and other self-inflicted injuries in observational analyses. J Clin Epidemiol.

[17] Rojas-Saunero LP, Hilal S, Murray EJ, Logan RW, Ikram MA, Swanson SA (2021). Hypothetical blood-pressure-lowering interventions and risk of stroke and dementia. Eur J Epidemiol.

[18] Dickerman BA, García-Albéniz X, Logan RW, Denaxas S, Hernán MA (2019). Avoidable flaws in observational analyses: an application to statins and cancer. Nat Med.

[19] Aris IM, Sarvet AL, Stensrud MJ, Neugebauer R, Li LJ, Hivert MF (2021). Young JG. Separating algorithms from questions and causal inference with unmeasured exposures: an application to birth Cohort studies of early body mass index rebound. Am J Epidemiol.

[20] Hernán MA, Taubman SL (2008). Does obesity shorten life? The importance of well-defined interventions to answer causal questions. Int J Obes (Lond).

[21] Katsoulis M, De Stavola B, Lai AG, Gomes M, Diaz-Ordaz K (2022). The authors respond. Epidemiology..

[22] Lévesque LE, Hanley JA, Kezouh A, Suissa S (2010). Problem of immortal time bias in cohort studies: example using statins for preventing progression of diabetes. BMJ..

[23] Maringe C, Majano SB, Exarchakou A, Smith M, Rachet B, Belot A (2020). Reflection on modern methods: trial emulation in the presence of immortal-time bias. Assessing the benefit of major surgery for elderly lung cancer patients using observational data. Int J Epidemiol.

[24] Wannamethee SG, Shaper AG, Walker M (2005). Overweight and obesity and weight change in middle aged men: impact on cardiovascular disease and diabetes. J Epidemiol Community Health.

[25] Alharbi TA, Paudel S, Gasevic D, Ryan J, Freak-Poli R, Owen AJ (2021). The association of weight change and all-cause mortality in older adults: a systematic review and meta-analysis. Age Ageing.

[26] Fridman M, Lucas ME, Paprocki Y, Dang-Tan T, Iyer NN (2020). Impact of weight change in adults with type 2 diabetes Mellitus: a literature review and critical analysis. Clinicoecon Outcomes Res.

[27] Jones NR, Taylor KS, Taylor CJ, Aveyard P (2019). Weight change and the risk of incident atrial fibrillation: a systematic review and meta-analysis. Heart..

[28] Hayes M, Baxter H, Müller-Nordhorn J, Hohls JK, Muckelbauer R (2017). The longitudinal association between weight change and health-related quality of life in adults and children: a systematic review. Obes Rev.

[29] Jung SJ, Woo HT, Cho S, Park K, Jeong S, Lee YJ (2017). Association between body size, weight change and depression: systematic review and meta-analysis. Br J Psychiatry.

[30] NCD Risk Factor Collaboration (NCD-RisC). Worldwide trends in body-mass index, underweight, overweight, and obesity from 1975 to 2016: a pooled analysis of 2416 population-based measurement studies in 128.9 million children, adolescents, and adults. Lancet. 2017;390:2627–42. 10.1016/S0140-6736(17)32129-3 PMC5735219 29029897

[31] Katsoulis M, Lai AG, Diaz-Ordaz K, Gomes M, Pasea L, Banerjee A (2021). Identifying adults at high-risk for change in weight and BMI in England: a longitudinal, large-scale, population-based cohort study using electronic health records. Lancet Diabetes Endocrinol.

[32] Peschanski N, Harouki N, Soulie M, Lachaux M, Nicol L, Remy-Jouet I (2021). Transient heart rate reduction improves acute decompensated heart failure-induced left ventricular and coronary dysfunction. ESC Heart Fail.

[33] Bianco A, Franco I, Osella AR, Giannelli G, Riezzo G, Bonfiglio C (2021). Physical activity reduction and the worsening of gastrointestinal health status during the second COVID-19 home confinement in Southern Italy. Int J Environ Res Public Health.

