# Supplementary material for: On the estimation of the effect of weight change on a health outcome using observational data, by utilising the target trial emulation framework
Source: Int J Obes (Lond). 2023 Oct 26;47(12):1309–17. doi: 10.1038/s41366-023-01396-0 (PMC10663146; doi:10.1038/s41366-023-01396-0)
Supplement: Supplementary file 1 — Appendix [file 41366_2023_1396_MOESM1_ESM.docx]

**APPENDIX**

**Section 1: Display items to highlight the problem of weight change on a health outcome**

**Table S1**: Data from weight measurements, confounder^*^ and outcome (CVD) needed to estimate the relationship of weight change in the first 2 years with CVD (from Table 1)

|  | **T=0** | **T=1** | **T=2** | **T=3** | **T=4** | **T=5** | **T=6** | **T=7** | **T=8** | **T=9** | **T=10** |
| --- | --- | --- | --- | --- | --- | --- | --- | --- | --- | --- | --- |
| **Years from time zero (enrolment)** | 0 | 2 | 4 | 6 | 8 | 10 | 12 | 14 | 16 | 18 | 20 |
| **Weight measurements** | B_0_ | B_1_ |  |  |  |  |  |  |  |  |  |
| **Confounder^*^** | C_0_ | C_1_ |  |  |  |  |  |  |  |  |  |
| **Allocation to an intervention A** |  | 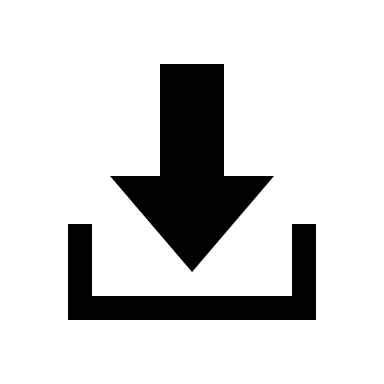 |  |  |  |  |  |  |  |  |  |
| **Outcome (CVD) measured^**^** | O_0_ | O_1_ | O_2_ | O_3_ | O_4_ | O_5_ | O_6_ | O_7_ | O_8_ | O_9_ | O_10_ |

^*^In table 1, there is only one confounder (chronic disease). Physical activity is another confounder, but all individuals have the same (high) levels of physical activity. In method 4, we exclude individuals with a chronic disease (in alliance with the eligibility criteria)

^*^Using method 4, we exclude individuals if they had prevalent CVD at enrolment (O_0_=1) or developed the outcome in the time until the 1^st^ follow-up (O_1_=1)

**Table S2**: Presentation of the data from the healthcare database from example (1) along with the estimated risk from each method

| **ID** | **number of individuals*** | **Intervention^‡^** | **BMI group** | **Weight change (in 2 years follow-up)** | **chronic disease (in 2 years follow-up) affecting weight change** | | **CVD (end of follow-up, i.e. at 20 years)** | **Used in method 1** | | **Used in method 2** | | **Used in method 3** | |  |  |  |
| --- | --- | --- | --- | --- | --- | --- | --- | --- | --- | --- | --- | --- | --- | --- | --- | --- |
| 1 | 1 | (a) | overweight | loss | yes | | yes | ● | | ◌ | | ◌ | |  |  |  |
| 2 | 1 | (a) | overweight | loss | yes | | no | ● | | ◌ | | ◌ | |  |  |  |
| 3-4 | 2 | (a) | overweight | loss | no | | yes | ● | | ● | | ● | |  |  |  |
| 5-20 | 16 | (a) | overweight | loss | no | | no | ● | | ● | | ● | |  |  |  |
| 21 | 1 | (b) | overweight | loss | yes | | yes | ● | | ◌ | | ◌ | |  |  |  |
| 22 | 1 | (b) | overweight | loss | yes | | no | ● | | ◌ | | ◌ | |  |  |  |
| 23-24 | 2 | (b) | overweight | remain | no | | yes | ● | | ● | | ● | |  |  |  |
| 25-40 | 16 | (b) | overweight | remain | no | | no | ● | | ● | | ● | |  |  |  |
|  | | | | | | | | | | | Estimation using method | | | | |  |
|  |  |  |  |  |  |  | | |  | | 1 | | 2 | | 3 | |
|  |  |  |  |  | **Overweight** | Risk weight loss | | | | | 4/22 | | 2/18 | | 2/18 | |
|  |  |  |  |  |  | Risk weight maintenance | | | | | 2/18 | | 2/18 | | 2/18 | |
|  |  |  |  |  |  | **Risk difference (%)** | | | | | **7.1%** | | **0%** | | **0%** | |

^*^As mentioned in the text, each of these individuals may represent millions (i.e. precision in the confidence interval is beyond the scope of this paper)

**^‡^**Interventions from the trials: (a) low caloric intake and high physical activity and

(b) standard caloric intake and high physical activity

**Table S3**: Problems in the interpretation of the findings when estimating the effect of (healthy) weight change on a health outcome using methods 1-3

|  | **Observational study** | | |
| --- | --- | --- | --- |
|  | **Methodology 1**  **(no adjustment for confounders at 1^st^ follow-up)** | **Methodology 2**  **(Follow-up starts after weight change)** | **Methodology 3**  **(proposed)** |
| **Problems in the interpretation** | 1. The intervention is very ill-defined, as no attempt has been made to distinguished intended from unintended weight change. As a result, the effect of weight change might be due to either the effect of (unknown) hypothetical interventions, e.g. physical activity, smoking cessation, diet, or due to a disease 2. Immortal time bias | 1. Ill-defined intervention, even if this problem is less pronounced compared to method 1 2. Immortal time bias 3. Follow-up time not correctly specified 4. Not appropriately emulating randomisation at time zero, as this method does not account for confounders at time zero. | 1. Ill-defined intervention, even if this problem is less pronounced compared to method 1 2. Immortal time bias |

**Figure S1**: Directed acyclic graph for the effect of weight change on CVD from table 1

v

C_0_  C_1_

B_0_ B_1_ O

A

Phase Phase

I II

0 2y (1^st^ follow-up) Time

**B_0_** is weight at time 0 and **B_1_** is weight at the 1^st^ follow-up (at 2y). **C_0_** are the confounders at time 0 and **C_1_** are the confounders at 2y and **O** is the outcome. Time is split into two phases. Phase I is between weight measurements at time 0 and the 1^st^ follow-up. Phase II is after the weight measurement at the 1^st^ follow-up. Weight change is only observed at the 1^st^ follow-up (end of Phase I), i.e. when we can measure **A=B_1_-B_0_**. **A** and **B_1_** can be used in the DAG interchangeably, as they are deterministically related (see red circle).  The arrow from B_1_ to A is deterministic and thus this DAG is collapsing to the following

v

C_0_  C_1_

B_0_ A O

Phase Phase

I II

0 2y (1^st^ follow-up) Time

Our aim is to find the effect of **A** (weight change) on the outcome **O.** From this DAG, we need to control/stratify for **C_0_,C_1_** and **B_0_**. If we do not control for **C_0_**, we leave the backdoor pathway A<--C_0_--> O open. In the same fashion, if we do not control for **C_1_**, we leave open the backdoor pathway A<--C_1_--> O open.

**Section 2: Simulation of the 2^nd^ dataset**

For the 2^nd^ dataset, we simulated data for 10,000 individuals.

We performed 1000 simulations. The simulation process is described below

Smoking status at time zero:

32% never smokers

20% former smokers

48% current smokers

Sex at time zero: ; variable name: sex

50% men

50% women

Age (in years) at time zero; variable name: age

Drawn from a normal distribution with: Mean: 50, sd: 2.5

Family history of CVD; variable name: fh_CVD

It is drawn from a Bernoulli distribution with

Pr(fh_CVD=1) = 0.12+ sd 0.05

Diuretics at time zero; variable name: diuretics0

It is drawn from a Bernoulli distribution with

Pr(diuretics0 =1) = -0.14+0.03*age+0.1*sex + sd 0.04

BMI at time zero; variable name: BMI0

Conditional on age, sex, family history of CVD, smoking status (at time zero) and diuretics (at time zero), BMI at time zero was drawn from a normal distribution with mean 25.8+0.06*(age-18)+0.1*sex+0.5*fh_CVD-0.1*sm_status0-0.2*diuretics0 and sd 0.56

Height: variable name: height

Drawn from a normal distribution with:

Mean: 1.63m, sd: 0.03m --> for women

Mean: 1.80m, sd: 0.03m --> for men

Smoking cessation between time zero and the 1^st^ follow-up; variable name: sm_cess1

It is equal to 0 for never and former smokers at time zero and for those who were current smokers at time zero, it is drawn from a Bernoulli distribution with

Pr(sm_cess=1) = -1.8+0.041*age-0.01*sex+0.2*diuretics0+ sd 0.04

Diuretics at the 1^st^ follow-up; variable name: diuretics1

It is drawn from a Bernoulli distribution with

Pr(diuretics1 =1) = 1.4+0.03*age+0.1*sex+0.1*diuretics0 + sd 0.04

CVD at the 1^st^ follow-up; variable name: CVD1

It is drawn from a Bernoulli distribution with

Pr(CVD1 =1) = -1.9+0.035*age+0.03*sex+0.3*fh_CVD+0.1*diuretics0 + sd 0.03

BMI at the 1^st^ follow-up; variable name: BMI1

Conditional on BMI at time zero, age, family history of CVD, smoking status (at time zero) and diuretics (at time zero), BMI at time zero was drawn from a normal distribution with mean

BMI0-0.9*CVD1+ 0.1* sm_status0 +2.5*sm_cess1-1.8*diuretics0-0.9*diuretics1+0.005*age and sd 1.4

Weight: variable name: weight0 (at time zero) and weight1 (at 1^st^ follow-up)

Weight0=BMI0*height^2^

Weight1=BMI1*height^2^

Combining information on weight1 and weight0, we created the 3 categories of weight change, i.e.

weight loss:

(weight1- weight0)/ weight0 < -5%

weight maintenance:

(weight1- weight0)/ weight0 ≥ -5% & (weight1- weight0)/ weight0 ≤ 5%

weight gain: (weight1- weight0)/ weight0 >5%

We then simulated time to event data for the next 18 years from a Weibull distribution with lambda 10^-4^ and gamma 1.1 and the following log hazard ratio for the covariates (Table S4), after deleting the participants that developed CVD between time zero and the 1^st^ follow-up.

The Stata script from the simulated study can be found online on github (<https://github.com/mkatsoulis82/On-the-estimation-of-the-effect-of-weight-change-on-a-health-outcome/blob/main/Simulation%20study>)

Table S4: Log-hazard ratios from the simulation study

|  | LogHR |
| --- | --- |
| Former vs never smoker | 1 |
| Current vs never smoker | 1.5 |
| Men vs Women | 0.2 |
| Age per year | 0.03 |
| Diuretics at time zero | 0.6 |
| Family history of CVD | 0.2 |
| BMI at baseline | 0.02 |
| Smoking cessation at first follow-up | -0.4 |
| Diuretics at first follow-up | 0.2 |
| weight loss vs weight maintenance | 0 |
| weight gain vs weight maintenance | 0.3 |

See below in Table S5 the summary data from the simulation of the second example

Table S5: Descriptive characteristics of 10,000 individuals at enrolment and at 1^st^ follow-up and CVD incidence risk at the end of follow-up from 1000 simulated datasets

| **At enrolment** |  |
| --- | --- |
| Body mass index (BMI) in kg/m^2^; mean (sd) | 27.7 (0.6) |
| Age in years; mean (sd) | 50.0 (2.5) |
| Sex  Men; (%)  Women; (%) | 50.0%  50.0% |
| Smoking status  Never; (%)  Former; (%)  Current; (%) | 30.0%  20.0%  48.0% |
| Diuretics; (%) | 15.2% |
| Family history of CVD; (%)  Height in meters; men: mean (sd)  Height in meters; women: mean (sd) | 12.0%  1.80 (0.03)  1.63 (0.03) |
| **At 1^st^ follow-up** |  |
| CVD events; (%) | 2.9% |
| weight change*  Loss (<5%)  Maintenance (≥ - 5% & ≤ 5%)  Gain (>5%) | 17.5%  57.5%  25.1% |
| Smoking cessation after enrolment*; N (%) | 13.3% |
| Diuretics*; N (%) | 16.8% |
| **End of follow-up** |  |
| CVD events | 9.9% |

**Section 3: Estimation of weighted Kaplan-Meier curves (using IPW)**

Steps

1. Fit a multinomial logistic regression model, weight change as dependent variable (in 3 categories: weight loss, maintenance and gain) and adjust for all potential confounders, both from the enrolment as well as from the follow-up.

2. Estimate the probability of being allocated to a particular weight change group and then calculate the inverse of this probability (i.e. the inverse probability of weighting or IPW).

3. Calculate a weighted Kaplan-Meier estimator, using as weights for each individual the IPW. Before producing the Kaplan-Meier curves, add a period in the beginning of the follow-up time equal to the duration of the baseline period (in our simulation study of section 2, this is 2 years) in which the risk is zero for all interventions in the baseline period.

If the IP weights are high, then the estimates will be biased. For this reason, we opt to calculate stabilised weights, see below:

1. Fit a multinomial logistic regression model, weight change as dependent variable (in 3 categories: weight loss, maintenance and gain) and adjust for all potential confounders, both from the enrolment as well as from the follow-up, and predict the conditional probability of weight loss, maintenance and gain [(pr(A/L); A= weight change group, L=confounders]

2. Fit a multinomial logistic regression model, weight change as dependent variable (in 3 categories: weight loss, maintenance and gain) without adjusting for any confounder and predict the conditional probability of weight loss, maintenance and gain [(pr(A); A= weight change group]

3. Generate stabilized weights as IPW=pr(A)/pr(A|L)

4. Calculate a weighted Kaplan-Meier estimator, using as weights for each individual the IPW. Before producing the Kaplan-Meier curves, add a period in the beginning of the follow-up time equal to the duration of the baseline period (in our simulation study of section 2, this is 2 years) in which the risk is zero for all interventions in the baseline period.

For more details, check the script in Stata for the estimation of weighted Kaplan-Meier curves (using IPW) in github from one simulated dataset (<https://github.com/mkatsoulis82/On-the-estimation-of-the-effect-of-weight-change-on-a-health-outcome/blob/main/Weighted%20Kaplan%20Meier%20curves>)

**Section 4: Estimation of incidence risk curves using the g-formula and pooled logistic regression**

Steps

1. Fit a pooled logistic model to estimate the conditional probability of developing the outcome given exposure, baseline covariates, and time of follow-up. Add product terms between weight change and time *t* of modelling period (beginning after the 1^st^ follow-up), as well as *t* squared and *t* cubic.

2, Estimate one minus this probability to calculate the probability of not developing the outcome.

3. Estimate the probability of remaining free of the event after a certain time at t for subjects through multiplying the model’s predicted values through time t.

4. Calculate conditional (adjusted) risk curves under the conditions of weight loss, maintenance and gain for each observed combination of values of the covariates at enrolment and the 1^st^ follow-up (one minus the probability of remaining free of the event after a certain time at t).

5. Predict the (counterfactual) risk at time t for each subject under weight loss, maintenance or gain, regardless of the subject’s exposure status.

6. Calculate the mean of the conditional risk under weight loss, maintenance or gain, over all subjects

For more details, check the script in Stata for the estimation of incidence risk curves using the g-formula in github from one simulated dataset (<https://github.com/mkatsoulis82/On-the-estimation-of-the-effect-of-weight-change-on-a-health-outcome/blob/main/Risk%20Curves%20using%20the%20g-formula>)
